# Supplementary material for: Allosteric modulation in monomers and oligomers of a G protein-coupled receptor
Source: eLife. 2016 May 6;5:e11685. doi: 10.7554/eLife.11685 (PMC4900804; doi:10.7554/eLife.11685)
Supplement: Figure 1—source data 1. — Panel C–Parametric values for the effect of gallamine on the rate of dissociation of [3H]QNB. Panel D–Parametric values for the effect of gallamine on the binding of [3H]NMS at equilibrium. Panels E–G–Parametric values for the effect of strychnine on the binding of [3H]NMS to oligomers and monomers. DOI: http://dx.doi.org/10.7554/eLife.11685.004 [file elife-11685-fig1-data1.docx]

**Figure 1-source data 1**

**(Figure 1-source data 1, *Panel* *B)*. Monomeric status of the purified M_2_ receptor after chemical cross-linking.** The area under the densitometric trace from each lane of western blots such as those illustrated in Figure 1B was estimated in three segments corresponding roughly to monomers (*a*, 40–75 kDa), dimers (*b*, 75–170 kDa), and tetramers or larger oligomers (*c*, 170–360 kDa). Each value was expressed as a percentage of the total area for that lane, and the values from three different blots were averaged to obtain the means (± S.E.M.) listed in the table.

|  | Intensity (*%*) | | |
| --- | --- | --- | --- |
| Cross-linking | *a* | *b* | *c* |
| None | 97 ± 1 | 2.6 ± 1.3 |  |
| BS^3^ | 83 ± 5 | 14 ± 7 | 3.5 ± 1.8 |

**(Figure 1-source data 1, *Panel* *C*). Parametric values for the effect of gallamine on the rate of dissociation of [^3^H]QNB.** Monomers of the M_2_ receptor were purified from *Sf*9 cells, and the rate constant for the dissociation of [^3^H]QNB was measured at 30 ºC in the absence of an allosteric ligand (*k*_0_) and at graded concentrations of gallamine (G) (*k*_obsd_). The dose-dependence of the ratio *k*_obsd_/*k*_0_ was analyzed in terms of Equation 2 (*n* = 1), and the parametric values are listed in the table. The data and the fitted curve are illustrated in Figure 1C. Also listed in the table are the parametric values from a previous study of the M_2_ receptor in *Sf*9 membranes (Equation 2, *n* = 2) (Shivnaraine et al., 2012); the corresponding fitted curve is shown by the dashed line in Figure 1C. The number of experiments is shown in parentheses.

| [^3^H]QNB |  |  |  |  | *k*_obsd_/*k*_0_ | | | |
| --- | --- | --- | --- | --- | --- | --- | --- | --- |
| (nM) *^a^* | log *K*_1_ | log *K*_2_­ | *n*_H(1)_ | *n*_H(2)_ | *F*_1_ | *F*_2_ | [G] = 0 | [G]→∞ |
|  |  |  |  |  |  |  |  |  |
| *Purified receptor* | | |  |  |  |  |  |  |
| 1.0 (5) | −4.12 ± 0.13 | — | 1.28 ± 0.42*^b^* | — | 1.00 | *c* | 0.99 ± 0.01 | 0.11 ± 0.08 |
|  | | |  |  |  |  |  |  |
| *Receptor in membranes* | | |  |  |  |  |  |  |
| 0.20 (4) | −5.99 ± 0.31 | −4.35 ± 0.03 | 0.97 ± 0.30 | 0.80 ± 0.14 | −1.51 | 2.51 ± 0.82 | 1.02 ± 0.04 | 0.00 ± 0.00 |
|  |  |  |  |  |  |  |  |  |

*^a^* The mean concentration of the probe in the experiments represented in the analysis (S.E.M./*μ* < 0.047).

*^b^* The sum of squares is not significantly larger with *n*_H_ fixed at 1 (*P* > 0.3).

*^c^* One class of sites is sufficient for Equation 2 to describe the data (*P* = 0.8).

**(Figure 1-source data 1, *Panel* *D*). Parametric values for the effect of gallamine on the binding of [^3^H]NMS at equilibrium.** Monomers of the M_2_ receptor were purified from *Sf*9 cells, and the binding of [^3^H]NMS was measured at graded concentrations of gallamine after equilibration of the reaction mixture for 21 h at 30 ºC. The data from 3 experiments were analyzed simultaneously in terms of Equation 2 (*n* = 1) to obtain the parametric values listed in the table. The data and the fitted curve are illustrated in Figure 1D. Also listed in the table are the parametric values from a previous study of the M_2_ receptor in detergent-solubilized extracts from *Sf*9 membranes (Equation 2, *n* = 3) (Shivnaraine et al., 2012); the corresponding fitted curve is shown by the dashed line in Figure 1D. The mean concentration of [^3^H]NMS in all such experiments with gallamine or strychnine was 10.1 ± 0.2 nM (*i.e.*, Figs. 1C, 1D, 1E-G, and 3F).

| log *K*_1_ | log *K*_2_­ | log *K*_3_­ | *n*_H(1)_ | *n*_H(2)_ | *n*_H(3)_ | *F*_1_ | *F*_2_ | *F*_3_ |
| --- | --- | --- | --- | --- | --- | --- | --- | --- |
|  | | |  |  |  |  |  |  |
| *Purified receptor* | | |  |  |  |  |  |  |
| −3.54 ± 0.03 | — | — | 1.00 ± 0.06 | — | — | 1.00 | *a* | *a* |
|  | | |  |  |  |  |  |  |
| *Receptor in unprocessed extracts* | | |  |  |  |  |  |  |
| −5.69 ± 0.26 | −4.59 ± 0.11 | −3.36 ± 0.21 | 1.06 ± 0.21 | 1.82 ± 0.49 | 1.25 ± 0.09 | 0.56 | −0.56 ± 0.18 | 1 |
|  |  |  |  |  |  |  |  |  |

*^a^* One class of sites is sufficient to describe the data.

**(Figure 1-source data 1, *Panels* *E–G*). Parametric values for the effect of strychnine on the binding of [^3^H]NMS to oligomers and monomers.** Strychnine and [^3^H]NMS were added either simultaneously or sequentially to preparations of the M_2_ receptor, as described in the legend to Figure S2. Binding of the radioligand at graded concentrations of strychnine was analyzed in terms of Equation 2 (*n* = 1 or 2) to obtain the parametric values listed in the table. Bell-shaped effects were described with two terms (*n* = 2), one for the ascending limb and one for the descending limb; strictly inhibitory effects were described with one term (*n* = 1). The number of experiments included in each analysis is shown in parentheses, and the data are shown in Figure 1E–G.

| Order of mixing | log *K*_1_ | log *K*_2_­ | | | *n*_H(1)_ | | *n*_H(2)_ | | *F*_1_ | | | *F*_2_ |
| --- | --- | --- | --- | --- | --- | --- | --- | --- | --- | --- | --- | --- |
|  |  |  | | |  | |  | |  | | |  |
| *Receptor extracted from porcine atria* | |  | | |  | |  | |  | | |  |
| Simultaneous (3) | −5.95 ± 0.10 | −3.70 ± 0.07 | | | 0.81 ± 0.06 | | 1.36 ± 0.39 | | −0.57 ± 0.09 | | | 1.57 |
| Strychnine, then NMS (3) | −6.03 ± 0.10 | −4.58 ± 0.08 | | | 0.70 ± 0.06 | | 1.21 ± 0.24 | | −1.00 | | | 2 |
| NMS, then strychnine (3) | −6.89 ± 0.09 | −4.34 ± 0.14 | | | 1.82 ± 0.89 | | 1 *^a^* | | — | | | *—* |
|  | |  | | |  | |  | |  | | |  |
| *Receptor purified from Sf9 cells* | |  | | |  | |  | |  | | |  |
| Simultaneous (3) | −3.62 ± 0.03 | — | | | 1.23 ± 0.07 | | — | | 1.00 | | |  |
| Strychnine, then NMS (3) | −4.46 ± 0.09 | — | | | 0.62 ± 0.07 | | — | | 1.00 | | |  |
| NMS, then strychnine (3) | −3.32 ± 0.05 | — | | | *b* | | — | | 1.00 | | |  |
|  | | |  |  | |  | |  | |  |  | |

*^a^* The value is defined by a shallow minimum in the sum of squares and was fixed accordingly.

*^b^* Not defined.

**References**

Shivnaraine, R. V., Huang, X. P., Seidenberg, M., Ellis, J., and Wells, J. W. 2012. Heterotropic cooperativity within and between protomers of an oligomeric M_2_ muscarinic receptor. *Biochemistry* **51:** 4518-4540.
