# Supplementary material for: Allosteric modulation in monomers and oligomers of a G protein-coupled receptor
Source: eLife. 2016 May 6;5:e11685. doi: 10.7554/eLife.11685 (PMC4900804; doi:10.7554/eLife.11685)
Supplement: Figure 1—source data 2. — DOI: http://dx.doi.org/10.7554/eLife.11685.005 [file elife-11685-fig1-data2.docx]

**Figure 1-source data 2**

**Data for Figure 1–figure supplement 1**

**Parametric values for the effect of strychnine on the binding of [^3^H]NMS and [^3^H]QNB to membrane-bound M_2_ receptor.** Strychnine and either [^3^H]NMS or [^3^H]QNB were added simultaneously to membranes prepared from CHO cells expressing the M_2_ receptor, and binding was measured after incubation of the reaction mixture for 3 h or 12 h at 30 °C. The data were analyzed in terms of Equation 2 (*n* = 1 or 2), with single values of *K_j_* or *n*_H(_*_j_*_)_ assigned as shown in the table to data acquired after 3 and 12 hours. The constraints were without appreciable effect on the sum of squares (*P* > 0.05). The parametric values are listed in the table, and the fitted curves are shown in Figure1-figure-supplement 1. The number of experiments is shown in parentheses.

| Incubation | log *K*_1_ | log *K*_2_­ | *n*_H(1)_ | *n*_H(2)_ | *F*_1_ | *F*_2_ |
| --- | --- | --- | --- | --- | --- | --- |
|  |  |  |  |  |  |  |
| *Strychnine and [^3^H]NMS* | | | | | | |
| 3 h (3) | −4.80 ± 0.11 | −3.37 ± 0.03 | 0.72 ± 0.06 | 1.44 ± 0.09 | −3.21 | 2.21 ± 0.16 |
| 12 h (2) |  |  | 0.98 ± 0.14 | 1.07 ± 0.08 |  |  |
|  |  |  |  |  |  |  |
| *Strychnine and [^3^H]QNB* | | | | | | |
| 3 h (3) | −4.12 ± 0.03 | — | 1.20 ± 0.07 | — | 1.00 |  |
| 12 h (2) | −3.74 ± 0.05 | — |  | — | 1.00 |  |
|  |  |  |  |  |  |  |
