# Supplementary material for: Allosteric modulation in monomers and oligomers of a G protein-coupled receptor
Source: eLife. 2016 May 6;5:e11685. doi: 10.7554/eLife.11685 (PMC4900804; doi:10.7554/eLife.11685)
Supplement: Figure 1—source data 3. — DOI: http://dx.doi.org/10.7554/eLife.11685.006 [file elife-11685-fig1-data3.docx]

**Figure 1-source data 3**

**Data for Figure 1-figure supplement 2**

**Rate constants for the simulated binding of strychnine and [^3^H]NMS according to Scheme S1.** The values listed in the table were substituted into Equations 13–17 to obtain the simulations illustrated in Figure 1-figure supplement 2. The value of *α* was taken as 100 throughout.

| Equilibrium dissociation constant | | |  | Rate constants | | |
| --- | --- | --- | --- | --- | --- | --- |
| Parameter | Value | |  | Parameter | Value | |
|  |  |  |  |  |  |  |
| *K*_L_ | 1.0 ×10^−8^  M | |  | *k*_−L_ | 0.02 min^−1^ | |
|  |  |  |  | *k*_+L_ | 2.0 × 10^6^ m^−1^ min^−1^ | |
|  |  |  |  |  |  |  |
| *K*_A_ | 1.0 × 10^−4^  M | |  | *k*_−A_ | 2 min^−1^ | |
|  |  |  |  | *k*_+A_ | 2.0 × 10^4^ m^−1^ min^−1^ | |
|  |  |  |  |  |  |  |
| *K*_AL_ | 1.0 × 10^−2^  M | |  | *k*_−AL_ | 2.0 × 10^−4^ min^−1^ | |
|  |  |  |  | *k*_+AL_ | 2.0 × 10^−2^ m^−1^ min^−1^ | |
|  |  | |  |  |  | |
