# Supplementary material for: Allosteric modulation in monomers and oligomers of a G protein-coupled receptor
Source: eLife. 2016 May 6;5:e11685. doi: 10.7554/eLife.11685 (PMC4900804; doi:10.7554/eLife.11685)
Supplement: Figure 2—source data 1. — Panel E–Levels of significance for ligand-dependent changes in the FRET efficiency of FlAsH-reacted mCh-M2-FCM. DOI: http://dx.doi.org/10.7554/eLife.11685.010 [file elife-11685-fig2-data1.docx]

**Figure 2-source data 1**

**(Figure 2-source data 1, *Panel A*). Time-resolved fluorescence and fluorescence anistoropy of eGFP and eGFP-tagged M_2_ receptors.** eGFP was fused to the *N*-termini of the wild-type M_2_ receptor (eGFP-M_2_) and a truncated mutant lacking the first 13 amino acids (eGFP-truncM_2_). The tagged receptors were expressed in CHO cells and solubilized in digitonin–cholate to obtain the samples used in the assays. In the case of eGFP-M_2_, the samples were supplemented with ligand as shown in the table. eGFP was purchased from Clontech and dissolved in the same buffer. The concentration of eGFP-tagged receptor was 3–10 nM, and that of eGFP was 3–10 nM. Time-resolved measurements of fluorescence emission and fluorescence anisotropy resulted in single exponentials that were analyzed according to Equations 4–9 to obtain the fluorescence lifetimes, rotational correlation times, and corresponding standard deviations listed in the table. Further details are described in Materials and Methods.

| Sample | Fluorescence lifetime  (*τ*, ns) | Rotational correlation time  (*φ*, ns) |
| --- | --- | --- |
|  |  |  |
| eGFP | 3.0 ± 0.1 | 18 ± 2 |
| eGFP-truncM_2_ | 2.6 ± 0.1 | 30 ± 5 |
| eGFP-M_2_ | 2.5 ± 0.1 | 55 ± 7 |
| eGFP-M_2_ + carbachol (1 mM) | 2.5 ± 0.1 | 43 ± 5 |
| eGFP-M_2_ + NMS (1 *μ*M) | 2.5 ± 0.1 | 44 ± 5 |
| eGFP-M_2_ + gallamine (10 mM) | 2.6 ± 0.1 | 40 ± 5 |
| eGFP-M_2_ + NMS (1 *μ*M) + gallamine (10 mM) | 2.6 ± 0.1 | 30 ± 5 |
|  |  | |

**(Figure 2-source data 1, *Panel E*). Levels of significance for ligand-dependent changes in the FRET efficiency of FlAsH-reacted mCh-M_2_-FCM.** The FRET efficiency (*E*_app_) of the sensor in CHO cells was measured in the absence and presence of ligands as described in the legend to Figure 2F. The mean changes in the FRET efficiency (∆*E*_app_ ± S.D.) were compared by means of the *t*-statistic to obtain the corresponding levels of significance (*P*) shown in the table. The number of cells is shown in parentheses. The levels of significance for the difference in *E*_app_ between the vacant and liganded receptor are as follows: NMS (1 *μ*M), < 0.001; gallamine (10 mM), 0.048; NMS (1 *μ*M) + gallamine (10 mM), 0.010; carbachol (1 mM), < 0.001; pilocarpine (1 mM), 0.032.

|  |  | Level of significance for differences in ∆*E*_app_ (*P*) | | | |
| --- | --- | --- | --- | --- | --- |
| Ligand | ∆*E*_app_ (*%*) | NMS | Gallamine | NMS + gallamine | Carbachol |
|  |  |  |  |  |  |
| Pilocarpine (19) | 7.3 ± 4.7 | < 0.001 | 0.010 | 0.93 | < 0.001 |
| Carbachol (19) | −21.6 ± 11.2 | < 0.001 | < 0.001 | < 0.001 |  |
| NMS + gallamine (18) | 8.2 ± 4.2 | < 0.001 | 0.020 |  |  |
| Gallamine (42) | 5.0 ± 2.0 | < 0.001 |  |  |  |
| NMS (26) | 20.2 ± 5.1 |  |  |  |  |
|  |  |  |  |  |  |
