# Supplementary material for: Allosteric modulation in monomers and oligomers of a G protein-coupled receptor
Source: eLife. 2016 May 6;5:e11685. doi: 10.7554/eLife.11685 (PMC4900804; doi:10.7554/eLife.11685)
Supplement: Figure 3—source data 1. — DOI: http://dx.doi.org/10.7554/eLife.11685.015 [file elife-11685-fig3-data1.docx]

**Figure 3-source data 1**

**(Figure 3-source data 1, *Panel D*). Levels of significance for ligand-dependent changes in the FRET efficiency of FlAsH-reacted mCh-M_2_-FCM and mCh-M_2_(D103A)-FCM.** The sensor (mCh-M_2_-FCM), the binding-deficient mutant (mCh-M_2_(D103A)-FCM), and the mutant plus the wild-type receptor (M_2_) were expressed or co-expressed in CHO cells, and the FRET efficiency (*E*_app_) was measured in the absence and presence of NMS (1*μ*M) as described in the legend to Figure 3. The mean changes in the FRET efficiency (∆*E*_app_ ± S.D.) were compared by means of the *t*-statistic to obtain the corresponding levels of significance (*P*) listed in the table. The number of cells is shown in parentheses. The level of significance for the difference in *E*_app_ between the vacant and NMS-liganded receptor was < 0.001 throughout.

|  |  | Level of significance for differences in ∆*E*_app_ (*P*) | |
| --- | --- | --- | --- |
|  | ∆*E*_app_ (*%*) | mCh-M_2_(D103A)-  FCM + M_2_ | mCh-M_2_(D103A)-FCM |
|  |  |  |  |
| mCh-M_2_-FCM (26) | 20.2 ± 5.3 | < 0.001 | < 0.001 |
| mCh-M_2_(D103A)-FCM (18) | 0.5 ± 0.6 | < 0.001 |  |
| mCh-M_2_(D103A)-FCM + M_2_ (42) | 9.5 ± 1.8 |  |  |
|  |  |  |  |
