# Supplementary material for: Allosteric modulation in monomers and oligomers of a G protein-coupled receptor
Source: eLife. 2016 May 6;5:e11685. doi: 10.7554/eLife.11685 (PMC4900804; doi:10.7554/eLife.11685)
Supplement: Figure 5—source data 1. — DOI: http://dx.doi.org/10.7554/eLife.11685.021 [file elife-11685-fig5-data1.docx]

**Figure 5-source data 1**

**Data for Figure 5–figure supplement 2**

**Distance between the *α*-carbon atoms of Tyr^177^ and Asn^419^ in crystal structures of the M_2_ receptor.** The values shown in the table also are plotted in Figure 5- figure supplement 2.

| Structure | C_α_-Y177–C_α_-N419  (Å) |
| --- | --- |
|  |  |
| 3UON.pdb (Haga et al., 2012) | 14.15 |
| 4MQS.pdb (Kruse et al., 2013) | 13.38 |
| 4MQT.pdb (Kruse et al., 2013) | 11.62 |
|  |  |

References

Haga, K., Kruse, A. C., Asada, H., Yurugi-Kobayashi, T., Shiroishi, M., Zhang, C., Weis, W. I., Okada, T., Kobilka, B. K., Haga, T., and Kobayashi, T. 2012. Structure of the human M2 muscarinic acetylcholine receptor bound to an antagonist. *Nature* **482:** 547-551.

Kruse, A. C., Ring, A. M., Manglik, A., Hu, J., Hu, K., Eitel, K., Hubner, H., Pardon, E., Valant, C., Sexton, P. M., Christopoulos, A., Felder, C. C., Gmeiner, P., Steyaert, J., Weis, W. I., Garcia, K. C., Wess, J., and Kobilka, B. K. 2013. Activation and allosteric modulation of a muscarinic acetylcholine receptor. *Nature* **504:** 101-106.
