# Supplementary material for: Allosteric modulation in monomers and oligomers of a G protein-coupled receptor
Source: eLife. 2016 May 6;5:e11685. doi: 10.7554/eLife.11685 (PMC4900804; doi:10.7554/eLife.11685)
Supplement: Figure 5—source data 2. — DOI: http://dx.doi.org/10.7554/eLife.11685.022 [file elife-11685-fig5-data2.docx]

**Figure 5-source data 2**

**Data for Figure 5–figure supplement 3**

**Mean distances (Å) between the *α*-carbon atoms of Tyr^177^ and Asn^419^ in the M_2_ receptor with different combinations of allosteric and orthosteric ligands.** The calculations were based on the structure of the receptor with iperoxo at the orthosteric site (4MQS).

|  |  | Allosteric site | | |
| --- | --- | --- | --- | --- |
|  |  | Vacant | Strychnine | Gallamine |
|  |  |  |  |  |
| Orthosteric site | Vacant | 11.4 | 11.3 | 11.5 |
|  | NMS | 10.9 | 10.1 | 11.5 |
|  | QNB | 11.2 | 11.5 | 11.1 |
|  |  |  |  |  |
