# Supplementary material for: Allosteric modulation in monomers and oligomers of a G protein-coupled receptor
Source: eLife. 2016 May 6;5:e11685. doi: 10.7554/eLife.11685 (PMC4900804; doi:10.7554/eLife.11685)
Supplement: Source code 1. — DOI: http://dx.doi.org/10.7554/eLife.11685.027 [file elife-11685-code1.docx]

**Equation 1:**

subroutine func04 (nob1,nob2,x,p,f)

c

c This subroutine computes the sum of two exponentials, each of which is

c defined by a rate constant (k) and the corresponding fraction of the total,

c time-sensitive amplitude (FR, where FR1+FR2=1). The y-intercept and the

c asymptote can be defined in absolute or relative terms, depending upon the

c value of parameter 6.

c

c Dimensioned arrays: p(np), parameters and constants (in this case, np=6);

c x(ny), independent variable (time); f(ny), computed values of the function.

c The arrays x and f are assumed here to contain more than one set of data;

c nob1 and nob2 are the first and last elements associated with the values in

c the array p(np).

c

c x(j) = TIME

c p(1) = k1

c p(2) = k2

c p(3) = FR2

c p(4) = Y (X = 0)

c p(5) = Y (X HIGH)

c p(6) = Y SWITCH 0, NO CONSTRAINT

c 1, p(4) = Y(XLO)/Y(XHI)

c 2, p(5) = Y(XHI)/Y(XLO)

c

dimension p(np),x(ny),f(ny)

c

ip6=ifix(p(6))

xk1=p(1)

xk2=p(2)

if (ip6.eq.0) then

yxlo=p(4)

yxhi=p(5)

else if (ip6.eq.1) then

yxhi=p(5)

yxlo=p(4)*yxhi

else if (ip6.eq.2) then

yxlo=p(4)

yxhi=p(5)*yxlo

else

print 42, ip6

42 format (' ERROR: ip6 =',i2,'. VALUE MUST BE 0/1/2.')

stop

end if

c

do 45 j=nob1,nob2

f1=(1.-p(3))*exp(-xk1*x(j))

f2=p(3)*exp(-xk2*x(j))

f(j)=(yxlo-yxhi)*(f1+f2)+yxhi

45 continue

c

return

end

**Equation 2:**

subroutine func15 (nob1,nob2,x,p,f)

c

c This subroutine computes the sum of three Hill terms, each of which is

c defined by an apparent affinity (K, in units of concentration), a Hill

c coefficient (NHILL) and the corresponding fraction of the total specific

c signal (FR, where FR1+FR2+FR3=1). The asymptotes are Y(X LOW) and

c Y(X HIGH).

c

c Dimensioned arrays: p(np), parameters (in this case, np=10);

c x(ny), independent variable (log concentration of ligand L);

c f(ny), computed values of the function.

c The arrays x and f are assumed here to contain more than one set of data;

c nob1 and nob2 are the first and last elements associated with the values

c in the array p(np).

c

c x(j) = log [L]

c p(1) = -log K1

c p(2) = -log K2

c p(3) = -log K3

c p(4) = NHILL1

c p(5) = NHILL2

c p(6) = NHILL3

c p(7) = FR(2)

c p(8) = FR(3)

c p(9) = Y (X LOW)

c p(10)= Y (X HIGH)

c

dimension p(np),x(ny),f(ny)

c

xk1=(10.**(-p(1)))**p(4)

xk2=(10.**(-p(2)))**p(5)

xk3=(10.**(-p(3)))**p(6)

do 155 j=nob1,nob2

x1=10.**(x(j))

f1=(1.-p(7)-p(8))*xk1/(xk1+x1**(p(4)))

f2= p(7) *xk2/(xk2+x1**(p(5)))

f3= p(8) *xk3/(xk3+x1**(p(6)))

f(j)=p(10)+(p(9)-p(10))*(f1+f2+f3)

155 continue

c

return

end

Equation 3:

subroutine func25 (nob1,nob2,x,p,f)

c

c This subroutine computes specific binding as the sum of three Hill terms

c superimposed on a sloping baseline. The latter is assumed to be a constant

c fraction of the concentration of unbound radioligand (NS). Each Hill term

c is defined by a Hill coefficient (nH), a corresponding value of x at

c half-maximal amplitude (K) and the fractional contribution to the total

c specific signal (FR, where FR1+FR2+FR3=1). The total specific signal is

c the total concentration of binding sites ([R]t). Other parameters allow

c the value of the function to be scaled and offset.

c

c Dimensioned arrays: p(np), parameters and constants (in this case, np=17);

c x(ny,2), independent variables for each of ny measured observations;

c f(ny), computed values of the function.

c The arrays x and f are assumed here to contain more than one set of data;

c nob1 and nob2 are the first and last elements associated with the values in

c the array p(np).

c There are two independent variables: an unlabelled ligand (A, x(j,1)),

c and the radiolabelled probe (P, x(j,2)). For the purposes of this equation,

c it is assumed that the unlabelled ligand is present only to define the

c level of non-specific binding. The concentration therefore must be zero or

c saturating (i.e., sufficient to block the specific binding of the probe);

c intermediate values cannot be accommodated.

c The default units of the dependent variable are dpm/mL. If the specific

c activity is set at 0.45045 (i.e., 1/2.22), the units of the dependent

c variable are pmol/L.

c

c The parameters and independent variables may enter the calculation on a

c linear (ln) or logarithmic (lg) scale, as determined by the values of

c parameters 15 and 16.

c The concentration of the probe can be taken as the free (i.e., unbound)

c concentration or the total (i.e., bound plus unbound) concentration, as

c determined by the value of parameter 17. Analyses in terms of the total

c concentration therefore will account for depletion of the probe through

c binding to the receptor.

c Parameters 15-17 are switches that direct the programme and therefore

c must not be optimised by the fitting routine.

c

c Further details regarding the numerical solution of this function have

c been described previously [Wells, J.W. (1992) 'Analysis and Interpretation

c of Binding at Equilibrium,' in 'Receptor-ligand Interactions. A Practical

c Approach,' Hulme, E.C. (ed.) Oxford University Press, pp 289-395].

c

c f(j) = TOTAL BINDING (dpm/mL)

c x(j,1) = [A] or log [A]

c x(j,2) = [P] or log [P]

c p(1) = K1 (mol/L) or pK1

c p(2) = K2 (mol/L) or pK2

c p(3) = K3 (mol/L) or pK3

c p(4) = nH1

c p(5) = nH2

c p(6) = nH3

c p(7) = FR(2)

c p(8) = FR(3)

c p(9) = [R]t (mol/L)

c p(10)= Rt SCALE

c p(11)= NS CONST

c p(12)= Y-OFFSET

c p(13)= Y-SCALE

c p(14)= SPECIFIC ACTIVITY (Ci/mmol)

c p(15)= PARAMETERS: ln/lg (1/2)

c p(16)= DATA: ln/lg (1/2)

c p(17)= FREE OR TOTAL LIGANDS (1/2)

c

dimension p(np),x(ny,2),f(ny)

common /aray25/ xp,r1t,r2t,r3t,xk1,xk2,xk3,xnh1,xnh2,xnh3

c

ip15=ifix(p(15))

ip16=ifix(p(16))

ip17=ifix(p(17))

xnh1=p(4)

xnh2=p(5)

xnh3=p(6)

c

if (ip15.eq.1) then

fac=(-alog(p(1))-alog(p(2))-alog(p(3)))/3.

fac=exp(fac)

xk1=(fac*p(1))**xnh1

xk2=(fac*p(2))**xnh2

xk3=(fac*p(3))**xnh3

else if (ip15.eq.2) then

fac=(p(1)+p(2)+p(3))/3.

fac=10.**fac

xk1=(fac*10.**(-p(1)))**xnh1

xk2=(fac*10.**(-p(2)))**xnh2

xk3=(fac*10.**(-p(3)))**xnh3

else

stop ' ERROR ON p(15). VALUE MUST BE 1 (LIN) OR 2 (LOG).'

end if

c

rt =fac*p(9)*p(10)

r1t=(1.-p(7)-p(8))*rt

r2t=p(7)*rt

r3t=p(8)*rt

c

if (ip17.eq.1) then

if (ip16.eq.1) then

do 252 j=nob1,nob2

if (x(j,1).gt.1.0e-10) then

xp=x(j,2)

pr=0.

else

xp=fac*x(j,2)

if (rt.eq.0.0.or.xp.eq.0.) then

pr=0.

else

if (r3t.eq.0.) then

f3=0.

else

xp3=xp**xnh3

xpk3=xp3/xk3

f3=r3t*(xpk3/(1.+xpk3))

end if

if (r2t.eq.0.) then

f2=0.

else

xp2=xp**xnh2

xpk2=xp2/xk2

f2=r2t*(xpk2/(1.+xpk2))

end if

if (r1t.eq.0.) then

f1=0.

else

xp1=xp**xnh1

xpk1=xp1/xk1

f1=r1t*(xpk1/(1.+xpk1))

end if

pr=f1+f2+f3

end if

pr=pr/fac

xp=xp/fac

end if

f(j)=((pr+p(11)*xp)*p(14)*2.22e12+p(12))*p(13)

252 continue

else if (ip16.eq.2) then

do 254 j=nob1,nob2

if (x(j,1).gt.-10.) then

xp=10.**x(j,2)

pr=0.

else

xp=fac*10.**x(j,2)

if (rt.eq.0.0) then

pr=0.

else

if (r3t.eq.0.) then

f3=0.

else

xp3=xp**xnh3

xpk3=xp3/xk3

f3=r3t*(xpk3/(1.+xpk3))

end if

if (r2t.eq.0.) then

f2=0.

else

xp2=xp**xnh2

xpk2=xp2/xk2

f2=r2t*(xpk2/(1.+xpk2))

end if

if (r1t.eq.0.) then

f1=0.

else

xp1=xp**xnh1

xpk1=xp1/xk1

f1=r1t*(xpk1/(1.+xpk1))

end if

pr=f1+f2+f3

end if

pr=pr/fac

xp=xp/fac

end if

f(j)=((pr+p(11)*xp)*p(14)*2.22e12+p(12))*p(13)

254 continue

else

stop ' ERROR ON p(16). VALUE MUST BE 1 (LIN) OR 2 (LOG).'

end if

else if (ip17.eq.2) then

if (ip16.eq.1) then

do 256 j=nob1,nob2

if (x(j,1).gt.1.0e-10) then

xp=x(j,2)

pr=0.

else

xp=fac*x(j,2)

if (rt.eq.0.0.or.xp.eq.0.) then

pr=0.

else

xmin=0.

xmax=rt

if (xp.lt.rt) xmax=xp

jj=j

pr=root25(jj,xmin,xmax)

end if

pr=pr/fac

xp=xp/fac

end if

f(j)=((pr+p(11)*(xp-pr))*p(14)*2.22e12+p(12))*p(13)

256 continue

else if (ip16.eq.2) then

do 258 j=nob1,nob2

if (x(j,1).gt.-10.) then

xp=10.**x(j,2)

pr=0.

else

xp=fac*10.**x(j,2)

if (rt.eq.0.0.or.xp.eq.0.) then

pr=0.

else

xmin=0.

xmax=rt

if (xp.lt.rt) xmax=xp

jj=j

pr=root25(jj,xmin,xmax)

end if

pr=pr/fac

xp=xp/fac

end if

f(j)=((pr+p(11)*(xp-pr))*p(14)*2.22e12+p(12))*p(13)

258 continue

else

stop ' ERROR ON p(16). VALUE MUST BE 1 (LIN) OR 2 (LOG).'

end if

else

stop ' ERROR ON p(17). VALUE MUST BE 1 (FREE) OR 2 (TOTAL).'

end if

c

return

end

c

c F25

c This subroutine computes the value of the function and the value

c of the first derivative of the function.

c

subroutine f25(x,fx,dfx)

common /aray25/ xp,r1t,r2t,r3t,xk1,xk2,xk3,xnh1,xnh2,xnh3

c

if (r3t.eq.0.) then

fx3=0.

dfx3=0.

else

xp3=(xp-x)**xnh3

xpk3=xp3/xk3

fx3=r3t*(xpk3/(1.+xpk3))

if (xp.ne.x.or.xnh3.ge.1.) then

dnum=xnh3*xk3*(xp-x)**(xnh3-1.)

dden=(xk3+xp3)**2

dfx3=r3t*dnum/dden

else

dfx3=1.e30

end if

end if

if (r2t.eq.0.) then

fx2=0.

dfx2=0.

else

xp2=(xp-x)**xnh2

xpk2=xp2/xk2

fx2=r2t*(xpk2/(1.+xpk2))

if (xp.ne.x.or.xnh2.ge.1.) then

dnum=xnh2*xk2*(xp-x)**(xnh2-1.)

dden=(xk2+xp2)**2

dfx2=r2t*dnum/dden

else

dfx2=1.e30

end if

end if

if (r1t.eq.0.) then

fx1=0.

dfx1=0.

else

xp1=(xp-x)**xnh1

xpk1=xp1/xk1

fx1=r1t*(xpk1/(1.+xpk1))

if (xp.ne.x.or.xnh1.ge.1.) then

dnum=xnh1*xk1*(xp-x)**(xnh1-1.)

dden=(xk1+xp1)**2

dfx1=r1t*dnum/dden

else

dfx1=1.e30

end if

end if

fx =-x+fx1+fx2+fx3

dfx=-1+dfx1+dfx2+dfx3

return

end

c

c ROOT25

c

c Using a combination of Newton-Raphson and bisection, this subroutine finds

c the root of the function f25 bracketed between X1 and X2. The root,

c returned as the function value ROOT25, will be refined until its accuracy

c is known within +/-EPS. F25 is the subroutine that returns both the

c function value and the first derivative of the function. (Adapted from

c Function rtsafe in Press et al. (1992) 'Numerical Recipies,' Cambridge

c University Press)

c

function root25(ipt,x1,x2)

c

parameter (maxit=100)

eps=0.00001

call f25(x1,fl,df)

call f25(x2,fh,df)

c

c ORIENT THE SEARCH SUCH THAT f(xl)<0

c

if (fl*fh.gt.0.) print 100, ipt

100 format (/,' WARNING: ROOT NOT BRACKETED AT POINT ',i3)

if (fl.lt.0.) then

xl=x1

xh=x2

else

xh=x1

xl=x2

swap=fl

fl=fh

fh=swap

end if

c

c INITIALISE THE GUESS FOR THE ROOT, THE 'STEP-SIZE BEFORE THE LAST',

c AND THE LAST STEP.

c

root25=0.5*(x1+x2)

dxold=abs(x2-x1)

dx=dxold

call f25(root25,f,df)

c

c LOOP OVER ALLOWED ITERATIONS

c

do 200 j=1,maxit

if (((root25-xh)*df-f)*((root25-xl)*df-f).ge.0.0.or.

* abs(2.*f).gt.abs(dxold*df)) then

dxold=dx

dx=0.5*(xh-xl)

root25=xl+dx

if (xl.eq.root25) return

else

dxold=dx

dx=f/df

temp=root25

root25=root25-dx

if (temp.eq.root25) return

end if

diff=(dxold-dx)/(dxold+dx)

test=abs(diff)

if (test.lt.eps) return

call f25(root25,f,df)

if (f.lt.0.) then

xl=root25

fl=f

else

xh=root25

fh=f

end if

200 continue

print 300, maxit

300 format (/,' root EXCEEDING MAXIMUM OF ',i3,' ITERATIONS.')

return

end
